# Finite element analysis of endoscopic cross-overtop decompression for single-segment lumbar spinal stenosis based on real clinical cases

**DOI:** 10.3389/fbioe.2024.1393005

**Published:** 2024-06-06

**Authors:** Yiwei Ding, Hanshuo Zhang, Qiang Jiang, Tusheng Li, Jiang Liu, Zhengcao Lu, Guangnan Yang, Hongpeng Cui, Fengtong Lou, Zhifeng Dong, Mei Shuai, Yu Ding

**Affiliations:** ^1^ School of Biological Science and Medical Engineering, Beihang University, Beijing, China; ^2^ Orthopedics, TCM Senior Department, The Sixth Medical Center of PLA General Hospital, Beijing, China; ^3^ Navy Clinical College, Anhui Medical University, Hefei, Anhui, China; ^4^ Chinese PLA Medical School, Beijing, China; ^5^ The Second School of Clinical Medicine, Southern Medical University, Guangzhou, Guangdong, China; ^6^ Department of Orthopedics, School of Medicine, South China University of Technology, Guangzhou, Guangdong, China; ^7^ Mechanical and Electronic Engineering Department, China University of Mining and Technology, Beijing, China

**Keywords:** lumbar spinal stenosis, endoscopic decompression, surgery, biomechanics, finite element analysis

## Abstract

**Introduction:** For severe degenerative lumbar spinal stenosis (DLSS), the conventional percutaneous endoscopic translaminar decompression (PEID) has some limitations. The modified PEID, Cross-Overtop decompression, ensures sufficient decompression without excessive damage to the facet joints and posterior complex integrity.

**Objectives:** To evaluate the biomechanical properties of Cross-Overtop and provide practical case validation for final decision-making in severe DLSS treatment.

**Methods:** A finite element (FE) model of L4-L5 (M0) was established, and the validity was verified against prior studies. Endo-ULBD (M1), Endo-LOVE (M2), and Cross-Overtop (M3) models were derived from M0 using the experimental protocol. L4-L5 segments in each model were evaluated for the range of motion (ROM) and disc Von Mises stress extremum. The real clinical Cross-Overtop model was constructed based on clinical CT images, disregarding paraspinal muscle influence. Subsequent validation using actual FE analysis results enhances the credibility of the preceding virtual FE analysis.

**Results:** Compared with M0, ROM in surgical models were less than 10°, and the growth rate of ROM ranged from 0.10% to 11.56%, while those of disc stress ranged from 0% to 15.75%. Compared with preoperative, the growth rate of ROM and disc stress were 2.66%–11.38% and 1.38%–9.51%, respectively. The ROM values in both virtual and actual models were less than 10°, verifying the affected segment stability after Cross-Overtop decompression.

**Conclusion:** Cross-Overtop, designed for fully expanding the central canal and contralateral recess, maximizing the integrity of the facet joints and posterior complex, does no significant effect on the affected segmental biomechanics and can be recommended as an effective endoscopic treatment for severe DLSS.

## 1 Introduction

Degenerative lumbar spinal stenosis (DLSS) is a prevalent debilitating condition among the elderly population, characterized by narrowing of the spinal canal and compression of the spinal cord and nerve roots, leading to symptoms such as lower back pain, numbness, tingling, weakness, particularly evident during standing or walking, and may be accompanied by intermittent claudication. It is typically caused by factors such as disc herniation, facet joint hypertrophy, ligamentum flavum thickening, and congenital bony spinal canal stenosis. When conservative treatment fails to provide relief, decompressive surgery to enlarge the spinal canal is often necessary (Yang et al., 2022). Open laminectomy decompression surgery can effectively decompress the canal but may cause significant damage to the posterior ligament complex and fail to maintain postoperative segmental stability, necessitating interbody fusion (Ouyang et al., 2023). However, the adjunctive internal fixation has drawbacks such as implant loosening, fusion cage subsidence, prolonged surgical time, and significant trauma, making it challenging for patients to tolerate (Hou et al., 2023). Advances in optical technology and surgical instruments have led to the expanding use of endoscopic spine surgery, offering improved clinical outcomes. Minimally invasive spine surgery (MISS) can effectively widen the spinal canal, alleviate neurological symptoms, reduce the risk of perioperative complications, sustain segmental biomechanical stability, and yield favorable surgical and rehabilitation outcomes (Zhang et al., 2021). Currently, both the two widely used coaxial endoscopy and unilateral biportal endoscopy (UBE) have their own advantages with good clinical efficacy. Compared to coaxial technique, UBE has demonstrated more flexible operations but requires larger incision and soft tissue injury (Yang et al., 2024). In addition, the latest concept of enhanced recovery after surgery (ERAS) has popularized MISS techniques for treating LSS, sparking professional interest in endoscopic spinal surgery (Kerolus et al., 2021).

**FIGURE 1:**
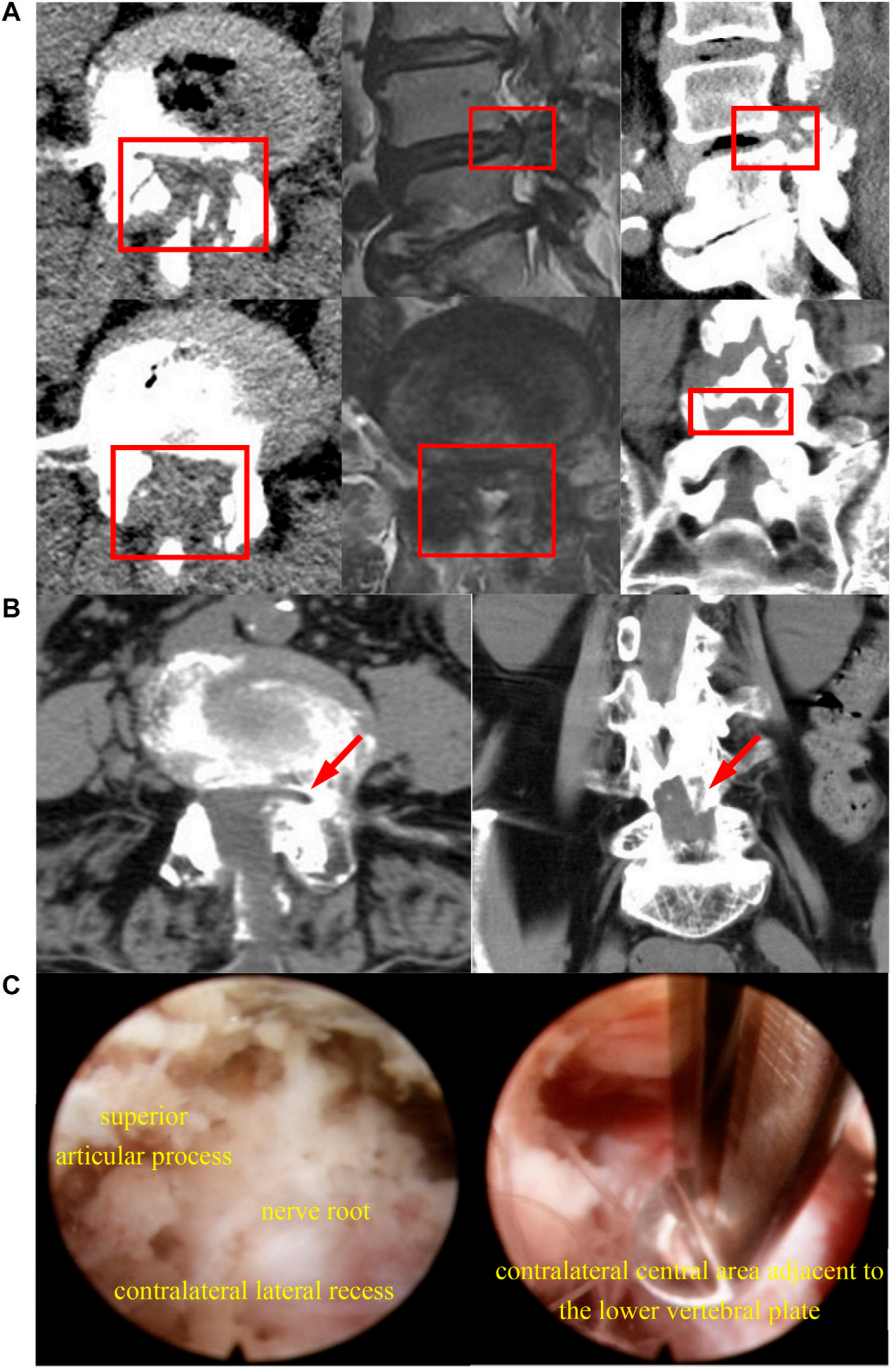
The diagram illustrates radiological and endoscopic images depicting inadequate decompression in the clinical application of the described techniques. In **(A)**, after bilateral Endo-LOVE surgery, though CT scans show sufficient osseous decompression, MRI images reveal suboptimal dural sac expansion at the sagittal region of the central lumbar canal, leading to unsatisfactory symptom relief clinically. **(B)** shows arrows indicating inadequate decompression of the contralateral lateral recess post-Endo-ULBD, failing to achieve satisfactory relief. **(C)** involves a repeated endoscopic observation on the opposite side of Endo-ULBD, with the upper image showing insufficient decompression of the contralateral recess and the lower image depicting central area inadequately decompressed.

Percutaneous endoscopic translaminar decompression (PEID), including classic operations such as unilateral laminotomy bilateral decompression (ULBD) and translaminar laminotomy decompression (Endo-LOVE), represents a prevailing and mature posterior technique in MISS treatment for DLSS(Algarni et al., 2023; Jiang et al., 2023; Li et al., 2023). However, Endo-ULBD and Endo-LOVE may encounter limitations when dealing with severe and intricate DLSS, i.e., the cauda equina convergence and epidural fat loss caused by the combination of small joint hyperplasia cohesion, ligamentum flavum hypertrophy and intervertebral foramen stenosis (Lee et al., 2010b; Lee et al., 2011; Park et al., 2013). Clinical experience indicates several imitations of Endo-ULBD, including insufficient intraoperative decompression of the central canal, contralateral recess issues, and certain endoscopic ‘blind areas’ in the contralateral field of view (Choi et al., 2016; Hua et al., 2020a; Hua et al., 2020b; Wu et al., 2021). Similarly, Endo-LOVE operations may exhibit inadequate central canal decompression for severe central canal stenosis, despite their effectiveness in addressing lateral canal and recess stenosis (Jiang et al., 2023). Figure 1, for example, illustrates that both Endo-ULBD and Endo-LOVE operations may not achieve optimal decompression clinically.

In light of these challenges, we introduced the modified endoscopically spinal canal decompression technique, named Cross-Overtop, involving bilateral crossed over-top trans-interlaminar laminotomy to enlarge the intersection of the narrow central canal and lateral recesses. The innovative Cross-Overtop operation essentially draws on the experiences of Endo-ULBD and Endo-LOVE operations and has the similarly basic surgical procedure, but mainly aims to enlarge the central spinal canal maximally while preserving the facet joints and posterior complex integrity as much as possible.

Based on Endo-LOVE and Endo-ULBD evaluation using the finite element (FE) lumbar spine model, we simulated the Cross-Overtop decompression range and performed the biomechanical analysis by overlapping and optimizing the first two surgical procedures. Furthermore, our research group applied the Cross-Overtop technique to severe DLSS clinical treatment in treating neuro-compressive symptoms. With the basic laminotomy and innovative overtop “undercutting” procedure in operation, Cross-Overtop decompresses the spine canal while preserving the midline structures and has shown to be clinically effective in treating central DLSS. In any case, surgical removal of the numerous ligamentum flavum and bony structures in Cross-Overtop may result in both positive and negative effects, such as spinal canal enlargement and possible segmental instability. Cross-Overtop focuses mainly on central canal decompression, so extensive resection of the posterior ligamentum flavum should be one of the critical factors for the lumbar biomechanical stability analysis. Although the posterior complex integrity, such as spinous process, interspinous and supraspinous ligaments, are well preserved in PEID operations, the possible excessive flexion of the lumbar spine following extensive ligamentum flavum resection in Cross-Overtop should be of great concern. Until now, no systematic FE analysis and biomechanical study of Cross-Overtop operation has been conducted, either theoretically or practically.

The innovation of this study is to explore the feasibility and rationality of Cross-Overtop decompression surgery from a biomechanical perspective and to provide a theoretical and practical basis for DLSS surgery decisions. Besides, the mutual verification of theory and practice is a brand-new research idea that will enrich and confirm the FE analysis in spine surgeries. The subsequent sections will provide a comprehensive description, including the experimental design, the establishment of finite element (FE) models based on virtual simulations and real clinical cases, the collection and statistical analysis of biomechanical assessment indicators, a detailed discussion of results, and the conclusive findings.

## 2 Materials and methods

### 2.1 Design

**FIGURE 2:**
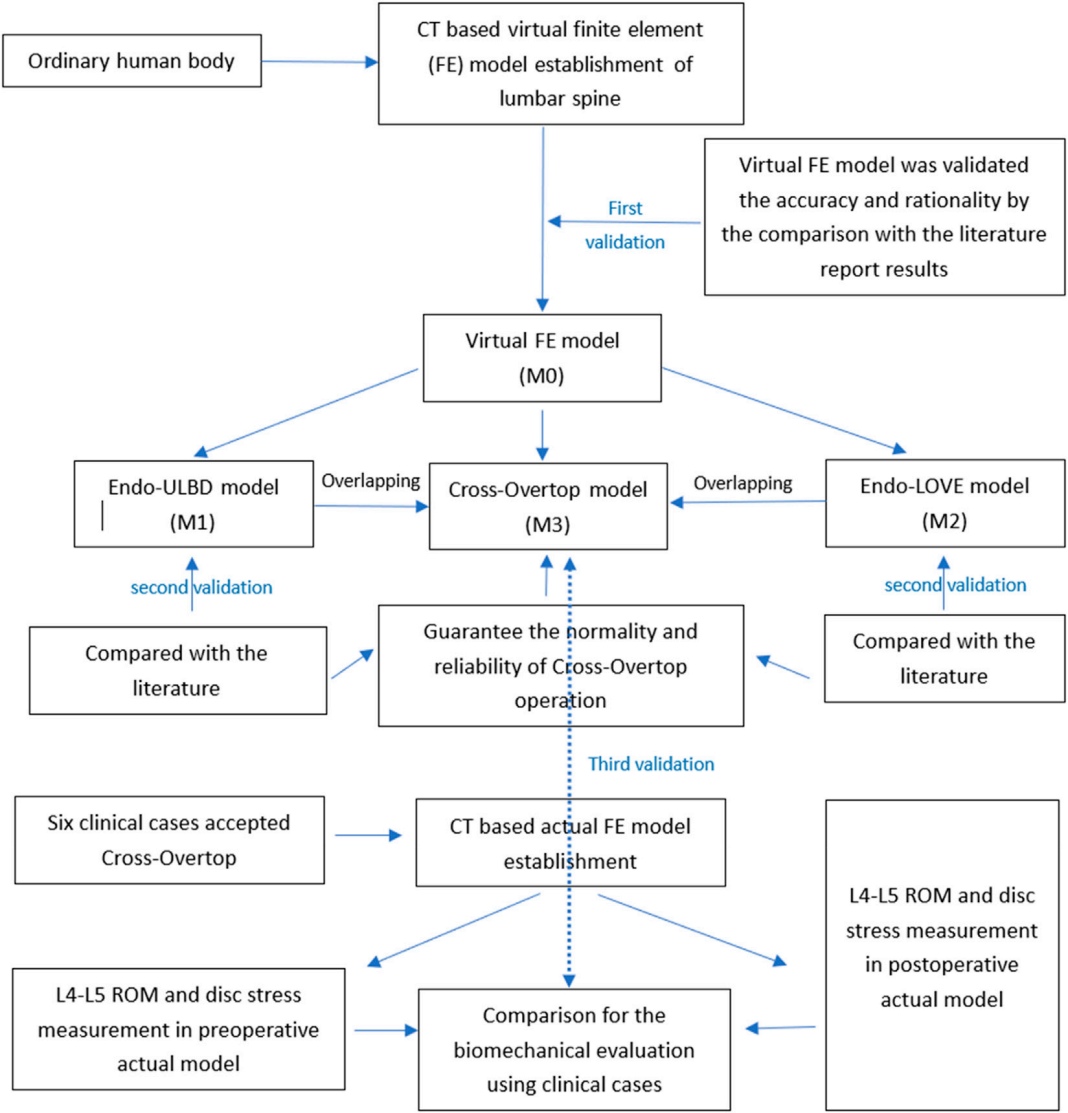
Flowchart illustrating the study design for the biomechanical evaluation of Cross-Overtop. The three-step validation, incorporating theory, practical application, and classical operations, enhances the credibility and reliability of the biomechanical analysis.

**FIGURE 3:**
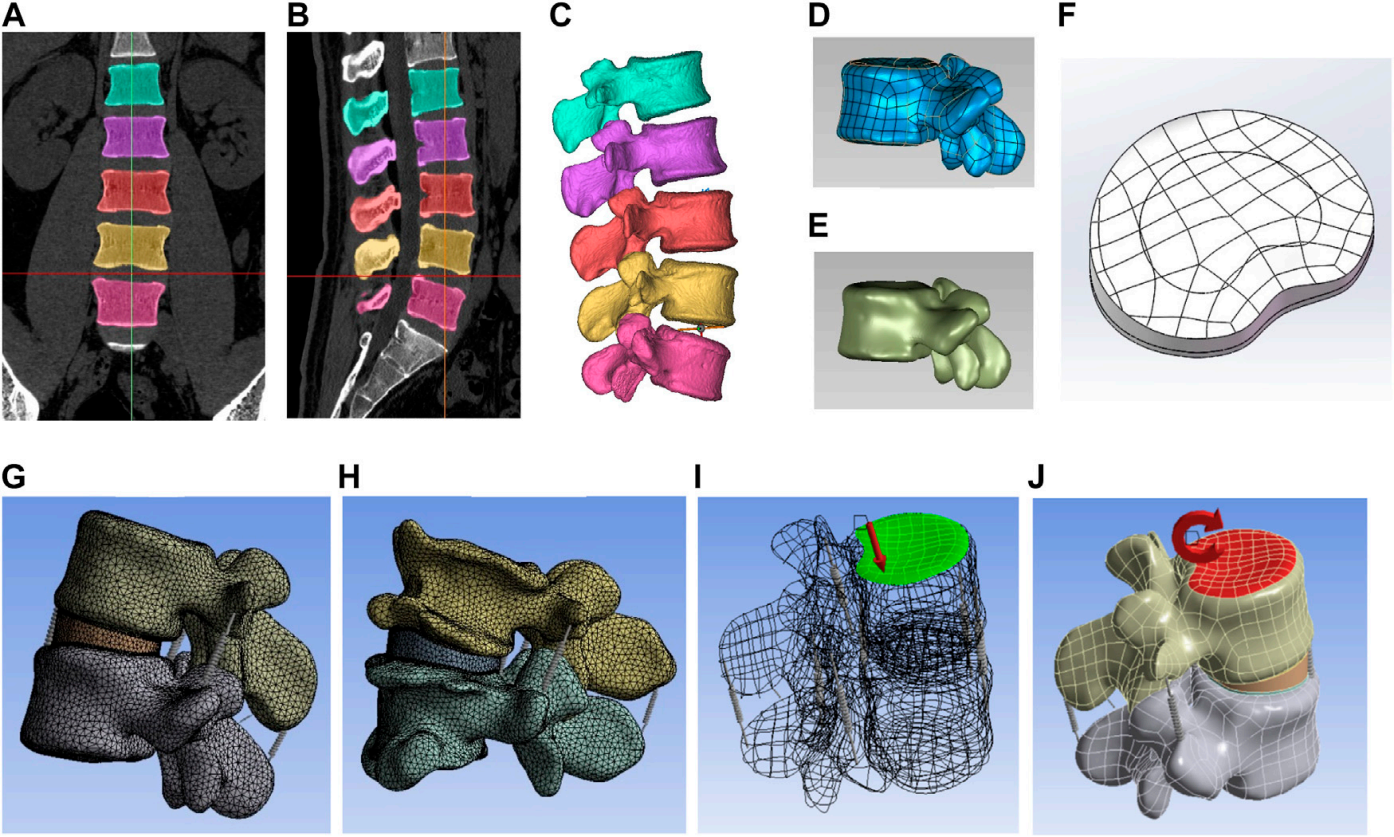
The diagram illustrates the three-dimensional FE analysis process. **(A,B)** depict the coronal and sagittal profiles of the lumbar spine, respectively. **(C)** demonstrates the construction of the mask to derive the preliminary bone structure. **(D)** exhibits the generation of a patch boundary structure comprising contour and boundary lines. **(E)** illustrates the generation of the NURBS surface fitting on the lumbar spine. **(F)** presents the developed superior endplate of the disc, along with the annulus fibrosus and nucleus pulposus. **(G)** displays the complete theoretical (virtual) model, showcasing the smooth surface of the disc and vertebral body. **(H)** reveals the full practical (actual) model, featuring the degenerative disc and hyperplastic vertebral body. **(I)** demonstrates the application of 500N pre-compressive loads on the superior surface of the L4 vertebra. **(J)** depicts segmental range of motion (ROM) tests conducted at the central top node of the L4 vertebra, applying 10 KN pure torque to simulate flexion, extension, left/right lateral bending, and axial rotation of the index segment.

An analysis of biomechanical compressive and tension strength was analyzed by the FE method, with observed indicators including segmental stability, range of motion (ROM), and disc stress. The study was structured into three steps. Initially, a theoretical FE model was constructed based on CT images of the ordinary human body. Subsequently, virtual simulation PEID surgeries, including Endo-ULBD, Endo-LOVE, and Cross-Overtop, were performed in the virtual FE model. The results of Endo-ULBD and Endo-LOVE confirmed operational specifications and test accuracy compared to literature reports, providing a foundation for the original Cross-Overtop decompression technique. Besides, we can make a horizontal comparison among the three PEID operations. Finally, actual lumbar FE models were developed based on the real clinical cases, which were used to simulate Cross-Overtop operation, and the analyzed results further confirmed the previous virtual research derivation. The designed three-step process involving multiple validations bolstered the credibility of biomechanical evaluation for the Cross-Overtop operation. Figure 2 shows the flowchart of the study design, and the FE analysis process is presented in Figure 3.

### 2.2 General information

Six patients diagnosed with DLSS undergone Cross-Overtop operation were recruited with the approval of the Ethics Committee (No. HZKY-PG-2021-14), and all patients signed an informed consent form as required. The virtual FE volunteer was an adult male without a lumbar disease history, and the patients participating in the clinical research suffered from severe DLSS. Successive lumbar spine scans were performed along the cross-section by Siemens dual-source spiral CT (Siemens AG, Munich, Germany). The CT images were divided into 473 layers and saved in DICOM format with a slice thickness of 0.625 mm. Cross-Overtop decompression has been progressively applied in clinical practice to improve low back pain, and neurologic deficits resulting from DLSS.

### 2.3 Software and equipment

Siemens 256-row helical CT (Siemens AG, Germany), Nicolet Viking Quest EMG/evoked (Natus, United States), Materialise Mimics 21 (Materialise NV, Belgium), Geomagic Wrap 2021 (3D Systems, United States), SolidWorks 2021 (Dassault Systèmes, French), ANSYS Workbench 17.0 (Ansys, United States) were used in the study.

### 2.4 Lumbar spine modeling with FE

Initially, CT image data of the L4-L5 segment was imported into Mimics for lumbar spine model creation using CT-based bone thresholding. Appropriate thresholding generated a lumbar spine mask by excluding soft tissue, followed by cavity filling and mask smoothing to reconstruct the initial L4-L5 3D model in STL format (Li et al., 2022b). Geomagic Studio was used for mesh optimization, encompassing feature removal, noise reduction, and other preprocessing steps to optimize the model structure (Ye et al., 2022). After employing “Grid Doctor” for validation and refinement, thin bone tissue was removed, and the “migration of the whole” technique established cancellous bone. Precise surface adjustments were performed, including manual contour editing, surface construction, and NURBS fitting.

Four lumbar vertebrae models were imported into SolidWorks and assembled using a fixed origin. The cancellous bone was meticulously positioned within the cortical bone, and any overlapping parts were meticulously removed to achieve the final assembly. Subsequently, the nucleus pulposus, annulus fibrosus, facet cartilage, and vertebral endplates were meticulously constructed through sketch drawing, stretching, and equidistant surface operations. Care was taken to ensure a nearly one-to-one correspondence between the nucleus pulposus and the annulus fibrosus within the intervertebral disc (Cao et al., 2020), and upon confirming interference corrections, a standard skeletal model was finalized.

The Ansys software facilitated model importation for component material parameterization, including elastic modulus and Poisson’s ratio, adhering to spinal structure material properties. The abnormal contact state was removed, and the correct connection quantity and relationship were confirmed, the facet joint was then regarded as contact friction with the coefficient set at 0.1, while the contact type of other parts was set as binding (Guo et al., 2020). In the connection option, the ligaments were simulated using Spring elements and represented by different stiffness values. The model finely meshed with the spring stiffness settings. Finally, the static structure was set with the fixed support, with the force and torque being loaded. The ligaments were adjusted after the various properties had been set. The geometry was then meshed in Ansys with specific parameters: 2.0 mm for cortical bone, 2.0 mm for cancellous bone, 1.0 mm for articular cartilage, 0.5 mm for upper and lower endplates, and 1.5 mm for both annulus fibrosus and nucleus pulposus. Once all properties were configured, the standard structure model M0 was generated. Table 1 shows the material parameters of the model components according to the material properties of the spinal structures based on the literature (Cao et al., 2020; Kahaer et al., 2023).

**TABLE 1:** Material parameters of lumbar FE model.

Items	Young’s modulus (MPa)	Poisson ratio
**Cortical bone [9]**	12,000	0.3
**Cancellous bone [9]**	100	0.2
**Endplate [9]**	24	0.4
**Annulus fibrosis [11]**	4.2	0.45
**Nucleus pulposus [11]**	1	0.499
**Anterior longitudinal ligament [9]**	20	0.3
**Posterior longitudinal ligament [9]**	70	0.3
**Ligamentum flavum [9]**	50	0.3
**Interspinous ligament [9]**	28	0.3
**Supraspinous ligament [9]**	28	0.3
**Intertransverse ligament [9]**	50	0.3

Note.–The material parameters of the model components are set according to the material properties of the spinal structures based on literature.

### 2.5 Force loading and motion simulation in lumbar spine model

The inferior surface of the L5 vertebra was immobilized during the simulation (Tan et al., 2021). 500N pre-compressive loads, equivalent to two-thirds of the body weight (75 kg), were applied on the superior surface of the L4 vertebra (Du et al., 2021). The segmental ROM tests were performed at the central top node of the L4 vertebra, exerting 10 KN pure torque to simulate flexion, extension, left-right lateral bending, and axial rotation of the index segment (Li et al., 2022a). The force and torque loading are presented in Figure 3 (part I and K). The ROM values were measured six times at different mark points, which were marked at the upper surface of L4 vertebrae to track the trajectory movement of models. The intervertebral disc maximum von Mises stresses were generated automatically via Ansys system software without manual measurement.

### 2.6 Clinical operation of Endo-ULBD, Endo-LOVE, cross-overtop, and establishment of simulation FE models

In the process of Endo-ULBD operation, the interlaminar plane of the surgical segment was located, and the borderline between the laminar margins and yellow ligament was determined. Initially, the interlaminar space was enlarged and part of the proliferative articular process was removed for the ipsilateral spinal canal and lateral recess decompression. Subsequently, the working cannula was tilted from the spinous process root to the contralateral spinal canal. For the contralateral canal decompression, the working area can reach the medial wall of the contralateral pedicle and the margin of the articular process, which facilitates the enlargement of the central spinal canal and the lateral lysis of the nerve roots (Kim et al., 2020).

For bilateral Endo-LOVE decompression, the lateral angle of interlaminar space was first identified. After partial lamina and articular process removal, the endoscopic working cannula was positioned to establish channels. Following initial lamina fenestration, the working channel head was placed between the intervertebral disc and ligament flavum for ventral-side dural sac and nerve root decompression. Then, the lateral recess was opened with the partial removal of the facet joint (Jiang et al., 2023). Ipsilateral Endo-LOVE completion was followed by contralateral decompression. Although the bilateral Endo-LOVE facilitated near-maximal completion of lumbar spinal canal decompression, the central canal decompression was generally insufficient.

**FIGURE 4:**
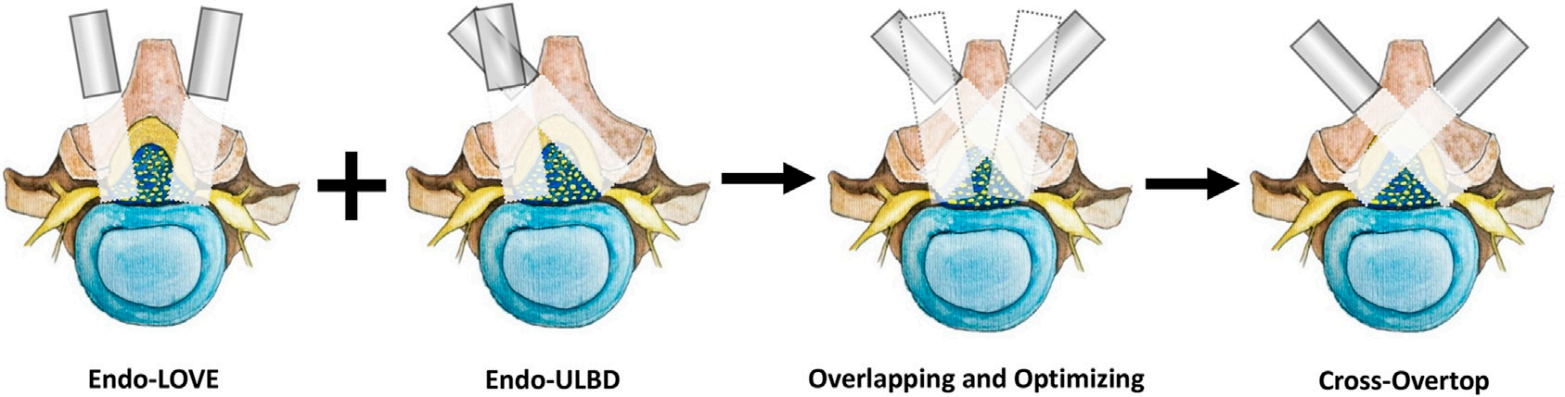
The diagram shows various PEID surgeries and the concurrent procedure for generating Cross-Overtop during FE analysis. During bilateral Endo-LOVE, the lamina fenestration and partial removal of facet joints are performed bilaterally. Although Endo-LOVE facilitated near-maximal completion of lumbar spinal canal decompression, the central canal was not sufficiently decompressed generally. For Endo-ULBD, the interlaminar space was enlarged, and sometimes, the excessive articular process should be removed ipsilaterally to achieve the overtop decompression. In the virtual FE model, the regularity and decompression range of the Cross-Overtop simulation are ensured by overlapping and optimizing Endo-LOVE and Endo-ULBD procedures. Through basic laminotomy and the overtop “undercutting” procedure, Cross-Overtop achieves maximum lumbar canal decompression while preserving the complex posterior integrity.

Cross-overtop integrated and refined the advantages of Endo-ULBD and Endo-LOVE to achieve a smaller range of facet removal and fuller spinal canal enlargement. Central and contralateral canal enlargement was performed by removing and penetrating the spinous process root in Cross-Overtop operation. Decompression spanned the dorsal central canal to the contralateral facet joint, repeated on the opposite side. The final Cross-Overtop operation was completed bilaterally, effectively eliminating the contralateral endoscopic blind area, ultimately expanding the spinal canal without undue sacrifice of the facet joints, and preserving the posterior complex integrity. Figure 4 illustrates the different PEID surgeries and the overlapping procedure to produce Cross-Overtop during FE analysis. In the virtual FE model, the regularity and decompression range of the Cross-Overtop simulation were ensured by overlapping and optimizing Endo-LOVE and Endo-ULBD procedures.

**FIGURE 5:**
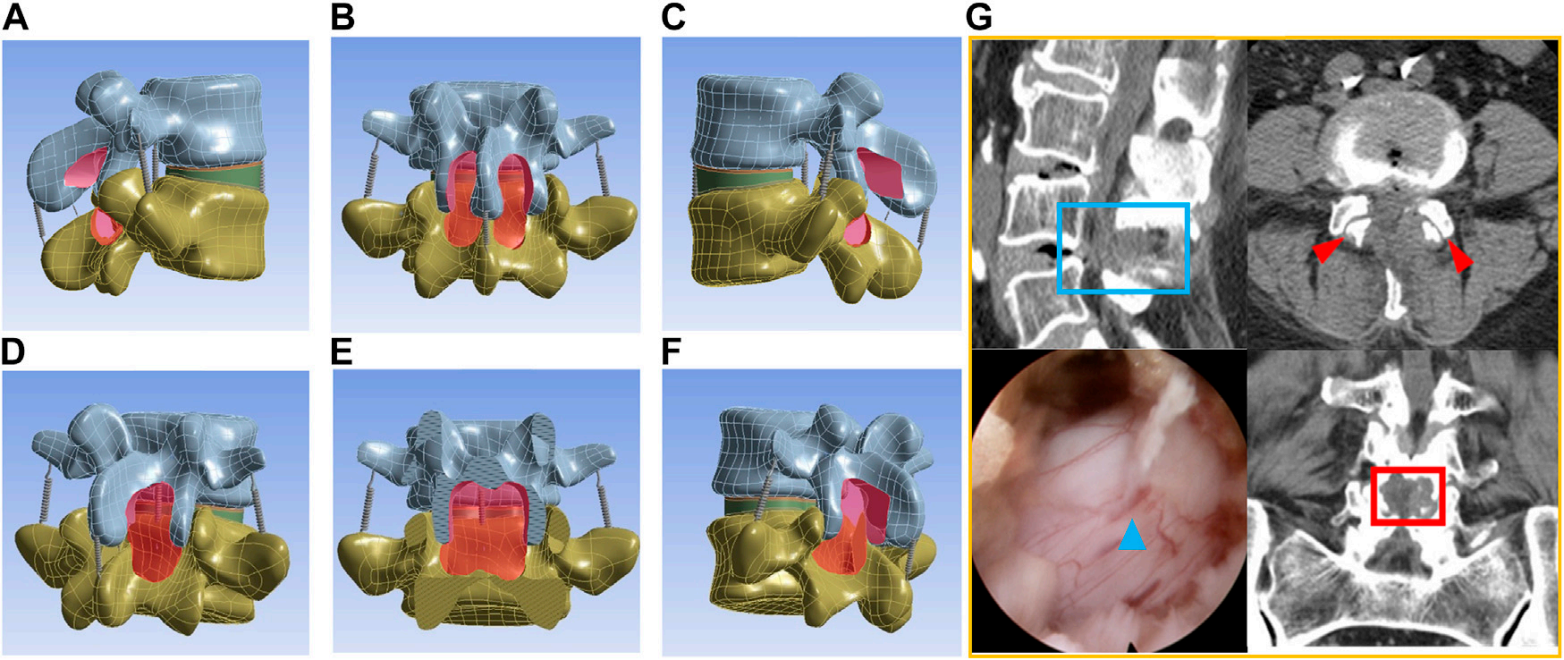
The diagram illustrates the decompression range of Cross-Overtop from various angles in FE models constructed from CT images. **(A,C)** (sagittal view) depict the partially resected root of the spinous process, while **(B,E)** (coronal view) illustrate laminotomy range extending upward to the laminar attachment of the ligamentum flava and downward to the lateral recess. In **(D,F)** (oblique sagittal view), the surface of the facet joint remains intact. **(G)** demonstrates the clinical application of the COTD. The blue boxes represent adequate decompression range in the sagittal view, while the red boxes indicate sufficient decompression in the coronal view. Blue triangles depict the postoperative dilation of the dural sac, and red triangles show ample preservation of facet joints. Cross-Overtop aims to maximize enlargement of the central spinal canal while preserving facet joints and posterior complex integrity to the greatest extent possible.

During the study, the above Endo-ULBD, Endo-LOVE, and Cross-Overtop operation simulations were performed in the virtual FE model. Based on the standard model M0, we obtained the Endo-ULBD model M1 by applying the convex stretching and segmenting of the sketch to excise part of the L4 spinous process root and using the endoscopic trephine saw to complete the multi-layer stereoscopic decompression. In Endo-LOVE model M2, the laminotomy and recess enlargement were performed side by side for the bilateral lumbar spinal canal decompression. In contrast, in Cross-Overtop model M3, we performed a limited overlapping of the Endo-ULBD and Endo-LOVE procedure with the bilateral crossed trans-interlaminar laminotomy for the intersection enlargement of the narrow central canal and lateral recesses. Figure 5 illustrates the decompression range of Cross-Overtop from various angles in the FE models developed from CT images. The depiction showcases the maximal enlargement of the central spinal canal while endeavoring to preserve the facet joints and maintain the integrity of the complex posterior structures.

**FIGURE 6:**
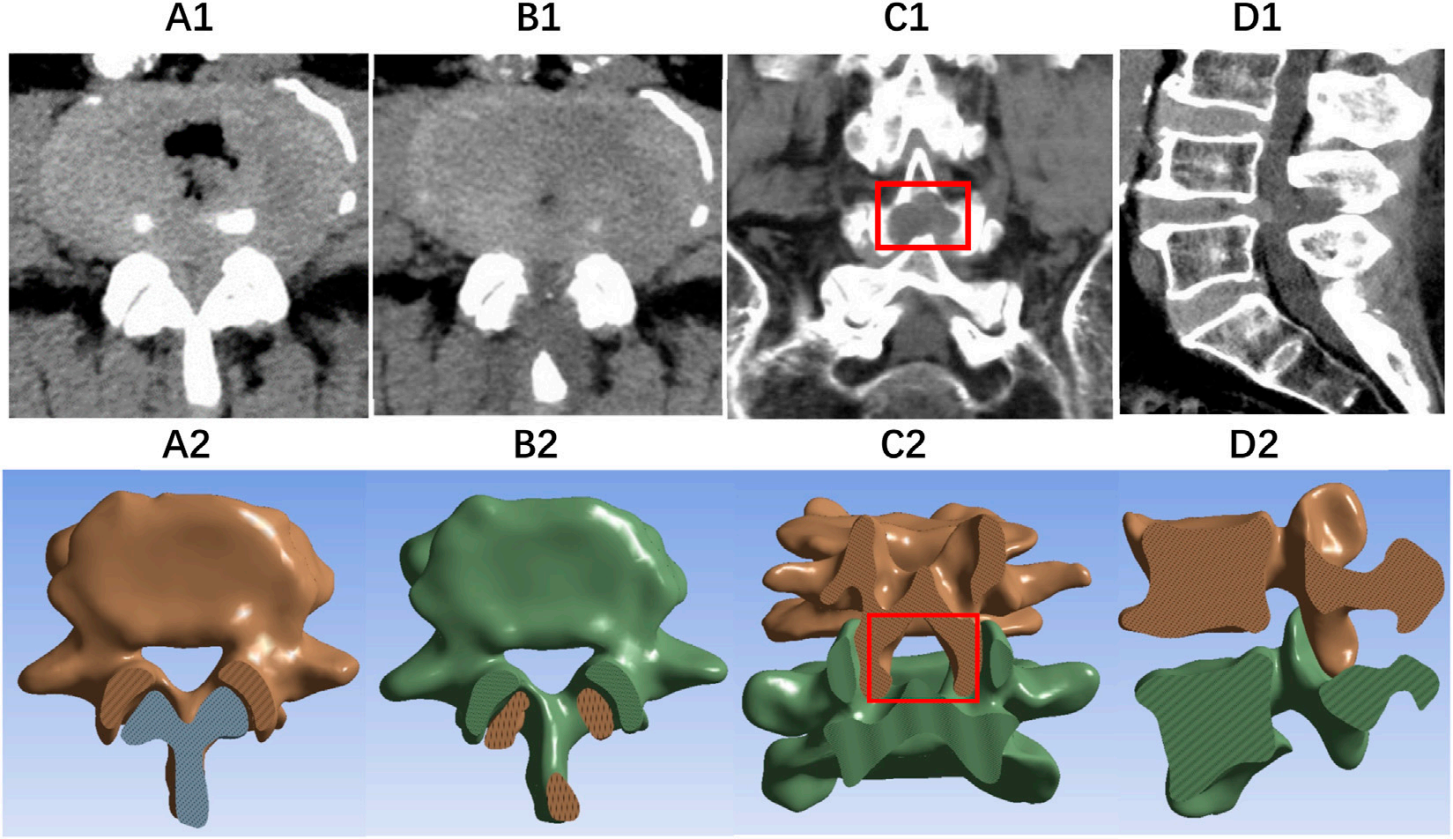
The diagram illustrates images of representative CT scans and the corresponding FE model establishment based on real clinical cases. **(A1, A2)** present the axial CT and FE images of DLSS, respectively. **(B1, B2)** display the axial plane, **(C1, C2)** show the coronal plane, and **(D1, D2)** provide the sagittal plane after Cross-Overtop, with well-preserved facet joints.

In a clinical setting, six patients with DLSS were enrolled following the Cross-Overtop operation. The practical data were extracted and modeled using the same steps as outlined in the earlier virtual modeling phase. Figure 6 illustrates the modeling of the actual scenario, presenting representative CT images and the corresponding Finite Element (FE) model established based on clinical cases. In the actual lumbar FE models, a 500N preload and 10 KN pure coupling torque were applied to the L4-L5 segment. Six physiological activity states were stimulated and regulated, covering flexion, extension, left-right bending, and rotation. The segmental ROM and disc stress were recorded and subjected to statistical analysis (Li et al., 2020).

### 2.7 Statistical analysis

We assessed ROM and disc stress under varied motion conditions. The ROM value was calculated as the mean and standard deviation of multiple measurements. The growth rate of ROM and disc stress were calculated as (postoperative mean value—preoperative mean value)/preoperative mean value x 100%. Because the FE models based on different CT images are not directly comparable even in the same case, the ROM and disc stress values cannot be compared statistically. Based on the literature, the growth rate of the observed parameters of less than 20% and the ROM values below 10° are regarded as the stable biomechanical state compared with M0 (Rao et al., 2002; Leone et al., 2007).

## 3 Results

### 3.1 Virtual model validation

**FIGURE 7:**
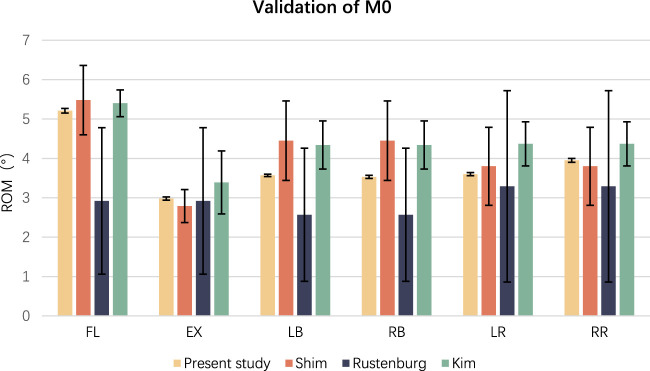
The diagram illustrates the ROM values across various studies. To validate the appropriateness of the proposed method, the virtual FE model was compared to previous experimental data. The ROM results are depicted in Figure 1, demonstrating a good fit between the model and experimental data. Consequently, M0 can reliably predict actual biomechanical responses in the spine.

The comparison between the parameters resulted from the established intact FE lumbar model, and the previous research (Shim et al., 2008; Kim et al., 2009; Rustenburg et al., 2019) revealed that the ROM of the M0 model was consistent with other scholars’ research results, indicating the FE model validity in this study. Table 2 presents overall alignment with prior values, ensuring the objectivity and subsequent experimental accuracy. The present study values are consistent with Shim’s research and have a constant tendency with Rustenburg’s and Kim’s analysis. Figure 7 demonstrates that M0 aligns well with the previous experimental data, validating its ability to predict credible biomechanical responses in the spine.

**TABLE 2:** Validation of M0 by ROM comparisons between the present and previous research (°).

Operating conditions in FE	Present research	Shim	Rustenburg	Kim
**Flexion**	5.21 ± 0.06	5.48 ± 0.88	2.92 ± 1.86	5.40 ± 0.34
**Extension**	2.98 ± 0.04	2.79 ± 0.42	2.92 ± 1.86	3.39 ± 0.80
**Left bending**	3.57 ± 0.03	4.45 ± 1.01	2.57 ± 1.69	4.34 ± 0.61
**Right bending**	3.53 ± 0.04	4.45 ± 1.01	2.57 ± 1.69	4.34 ± 0.61
**Left rotation**	3.60 ± 0.04	3.80 ± 0.99	3.29 ± 2.43	4.37 ± 0.56
**Right rotation**	3.95 ± 0.05	3.80 ± 0.99	3.29 ± 2.43	4.37 ± 0.56

Note.–The table presents the ROM, values in the standard FE, model (M0) and the comparison with the previous experimental data, which indicates the overall consistency between the present study values and previous research results and guarantees the objectivity and accuracy of the subsequent experimental research. The present study values are consistent with Shim’s study and have a constant tendency with Rustenburg’s and Kim’s research.

### 3.2 Measurement and analysis of ROM and disc stress in virtual models

**FIGURE 8:**
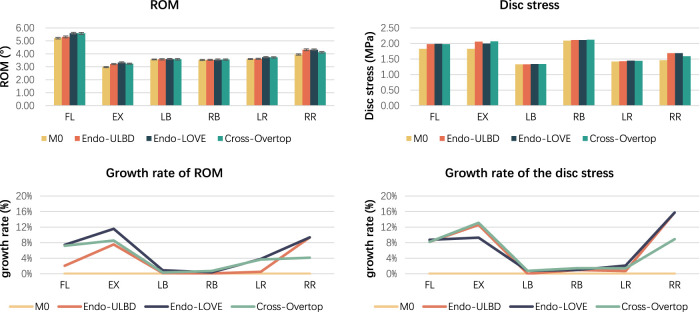
Histogram shows the values and growth rate of ROM and disc stress. All the parameters in the three surgical groups are within the stable range, as reported in the literature. The study’s segmental stability of Endo-ULBD and Endo-LOVE indicates the normalized simulated operation in the FE analysis. Also, Cross-Overtop is proven safe and reliable by the segmental stability test.

**FIGURE 9:**
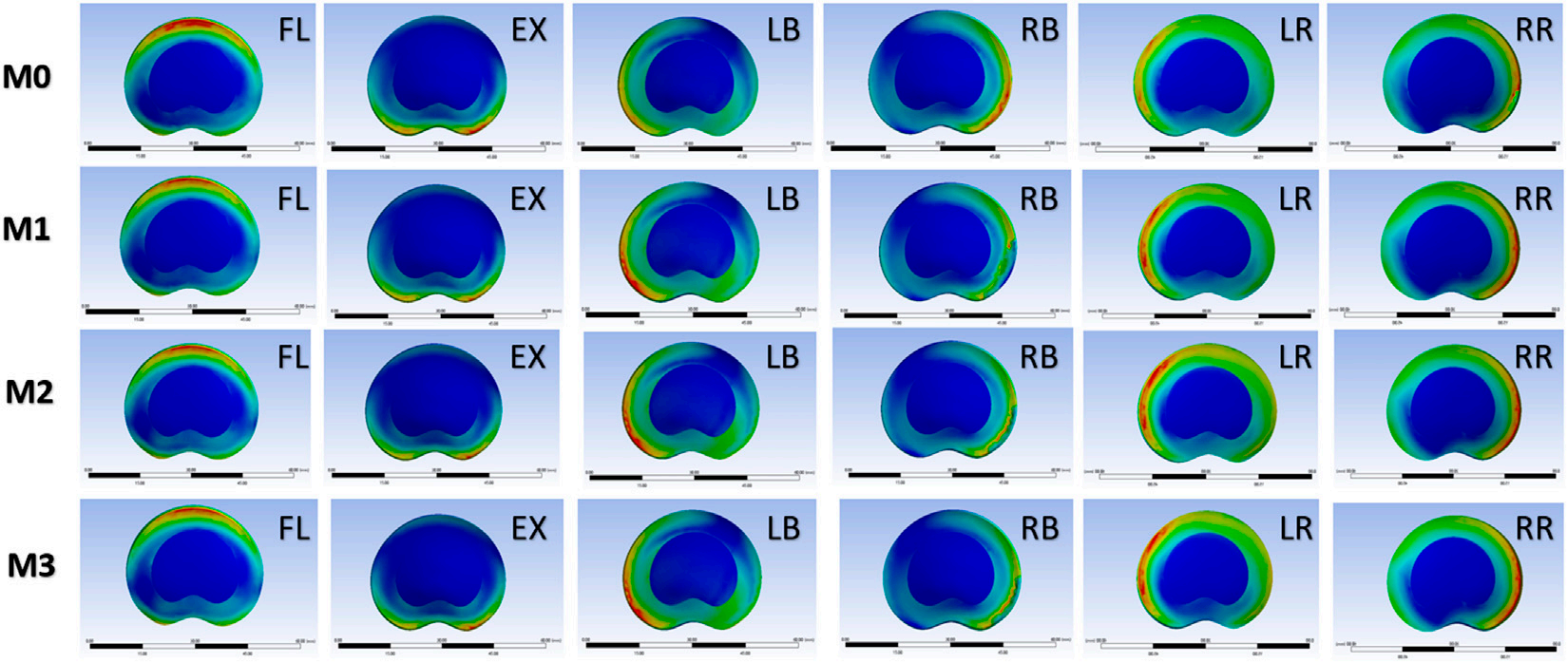
Diagram of equivalent disc stress cloud under six conditions, including flexion (FL), extension (EX), left bending (LB), right bending (RB), left rotation (LR), and right rotation (RR). Compared with M0, all three models present a uniform distribution of disc stress with no abnormal bulge in the cloud. M0 (standard model), M1 (Endo-ULBD model), M2 (Endo-LOVE model), M3 (Cross-Overtop model).

Under the six motion conditions of flexion, extension, left/right bending, and left/right rotation at the L4-L5 segment in the virtual models (M0, M1, M2, M3), the ROM and Von Mises stress of the disc were measured and analyzed respectively. The values in the four models and the growth rate compared with M0 are presented in Table 3. All the ROM values were less than 10°. The growth rate of ROM ranged from 0.10% to 11.56%, while those of disc stress ranged from 0% to 15.75%. Figure 8 presents the values and the growth rate of ROM and disc stress in the virtual FE model. All the observed parameters in the three surgical groups were within the stable range. The study’s segmental stability of Endo-ULBD and Endo-LOVE indicated the normalized simulated operation in the FE analysis. Additionally, Cross-Overtop was proved safe and reliable by the stability test at the affected segment. Figure 9 displays the equivalent disc stress clouds under six conditions. All three surgical models presented uniform disc stress distribution without sudden intensification or abnormal bulge in the diagram.

**TABLE 3:** ROM (degree), disc stress (MPa), and growth rate (%) of the observed biomechanical parameters among Endo-ULBD, Endo-LOVE, and Cross-Overtop in the virtual lumbar FE model.

Conditions in FE		Virtual lumbar (M0)		Endo-ULBD (M1)		Endo-LOVE (M2)		Cross-overtop (M3)	
		ROM	Disc stress	ROM	Disc stress	ROM	Disc stress	ROM	Disc stress
**Flexion**	**Value**	5.21 ± 0.06	1.83	5.31 ± 0.07	1.98	5.59 ± 0.07	1.99	5.58 ± 0.07	1.98
	**Growth rate**	—	—	2.05%	8.20%	7.43%	8.74%	7.20%	8.20%
**Extension**	**Value**	2.98 ± 0.04	1.83	3.21 ± 0.04	2.06	3.33 ± 0.05	2.00	3.24 ± 0.04	2.07
	**Growth rate**	—	—	7.54%	12.57%	11.56%	9.29%	8.55%	13.11%
**Left bending**	**Value**	3.57 ± 0.03	1.33	3.58 ± 0.05	1.33	3.60 ± 0.05	1.34	3.58 ± 0.06	1.34
	**Growth rate**	—	—	0.19%	0.00%	0.89%	0.75%	0.33%	0.75%
**Right bending**	**Value**	3.53 ± 0.04	2.09	3.54 ± 0.04	2.11	3.55 ± 0.06	2.11	3.56 ± 0.05	2.12
	**Growth rate**	—	—	0.10%	0.96%	0.38%	0.96%	0.71%	1.44%
**Left rotation**	**Value**	3.60 ± 0.04	1.42	3.62 ± 0.04	1.43	3.74 ± 0.05	1.45	3.73 ± 0.05	1.44
	**Growth rate**	—	—	0.51%	0.70%	3.80%	2.11%	3.66%	1.41%
**Right rotation**	**Value**	3.95 ± 0.05	1.46	4.32 ± 0.07	1.69	4.32 ± 0.06	1.69	4.12 ± 0.06	1.59
	**Growth rate**	—	—	9.36%	15.75%	9.36%	15.75%	4.13%	8.90%

Note.–The growth rate of ROM, and disc stress were calculated as (postoperative mean value - preoperative mean value)/preoperative mean value x 100%. ROM, value is described as mean ± standard deviation, with the unit as a degree. The maximum von Mises stress value (MPa) is generated automatically via Ansys system software without manual measurement, describing it in simple value instead of statistical calculation. Compared with M0, the growth rate of the observed biomechanical parameters of less than 20% and the ROM, value of less than 10° are regarded as the stable biomechanical state in the surgical models.

### 3.3 Measurement and analysis of ROM and disc stress in the actual model before and after cross-overtop

**FIGURE 10:**
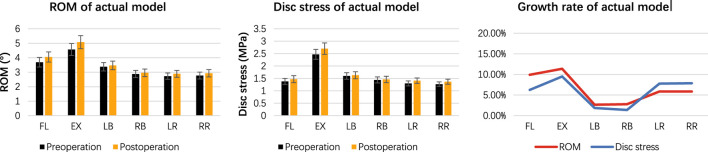
The histogram and line chart depict the ROM, disc stress, and related growth rates in the actual Cross-Overtop FE model. The ROM values are less than 10°, and the growth rates of ROM and disc stress are below 20%. Cross-Overtop does not have a noticeable effect on the affected segmental biomechanics.

The ROM, disc stress, and related growth rate under the actual Cross-Overtop FE model are shown in Figure 10. All the ROM values are less than 10°. Compared with the preoperative values, the growth rate of ROM and disc stress range from 2.66% to 11.38%, and 1.38%–9.51%, respectively. The results in the actual FE analysis, which presented a high consistency with those in the previous virtual study, verified the stability of the affected segment after the Cross-Overtop operation. Table 4 shows the detailed values of the observed parameters at the L4-L5 segment in the actual Cross-Overtop model.

**TABLE 4:** ROM (degree) and disc stress (MPa) of pre- and post-operation in actual Cross-Overtop FE mode (%).

Different conditions in FE	ROM			Disc stress		
	Preoperation	Postoperation	Growth rate (%)	Preoperation	Postoperation	Growth rate (%)
**Flexion**	3.69 ± 0.33	4.05 ± 0.35	9.93	1.38 ± 0.12	1.47 ± 0.14	6.25
**Extension**	4.57 ± 0.41	5.08 ± 0.45	11.38	2.47 ± 0.20	2.70 ± 0.23	9.51
**Left bending**	3.38 ± 0.29	3.47 ± 0.30	2.66	1.60 ± 0.13	1.63 ± 0.14	1.87
**Right bending**	2.88 ± 0.25	2.96 ± 0.26	2.78	1.44 ± 0.12	1.46 ± 0.13	1.38
**Left rotation**	2.73 ± 0.22	2.89 ± 0.24	5.87	1.30 ± 0.10	1.41 ± 0.11	7.78
**Right rotation**	2.77 ± 0.24	2.93 ± 0.25	5.85	1.27 ± 0.09	1.37 ± 0.10	7.88

Note.–Six patients were recruited, and the ROM, and disc stress values are described as mean ± standard deviation. All the ROM, values are less than 10°. Compared with the preoperative values, the growth rate of ROM, and disc stress range from 2.66% to 11.38%, and 1.38%–9.51%, respectively. The results indicate the stability of the affected segment after the Cross-Overtop operation.

## 4 Discussion

Decompression laminectomy leads to the destabilization of the affected lumbar segment due to the removal of posterior anatomical integrity. Resecting the facet capsule, posterior bony, and ligamentous elements of the lumbar spine reduces the segmental stability and may increase the deformation and loads at the index level (Spina et al., 2021). Open laminectomy combined with instrumented fusion was considered as the “criterion standard” treatment for DLSS. However, open spine surgery has several adverse effects, such as iatrogenic spinal instability, adjacent segmental degeneration, and secondary chronic low back pain (Overdevest et al., 2015; Spina et al., 2021). Mono- or bilateral laminotomy may be suggested for the expected relief of neurologic symptoms without apparent influence on the segmental stability, but conventional laminotomy has some decompressive deficiencies, especially for the severe and central lumbar stenosis due to the limitation of open surgery.

With the development of modern imaging technologies and the optimization of surgical instruments, endoscopic MISS techniques have been significantly improved for the treatment of degenerative spinal diseases. In recent decades, MISS has been widely accepted with various innovative methods. A proper method of sufficient decompression while rationally protecting the bony and ligamentous lumbar structures can be beneficial to prevent the possible secondary operation or internal fixation fusion in the future. Although literature reports have shown the satisfactory clinical effect of PEID surgery in treating DLSS, there are also numerous concerns and potential complications.

The classical LOVE surgery involves laminotomy combined with ligamentum flavum-disc space decompression and discectomy, with the full endoscopic operation referred to as lumbar laminotomy decompression or Endo-LOVE (Jiang et al., 2023; Zhang et al., 2023) Laminotomy, considered more delicate and associated with less iatrogenic instability compared to conventional laminectomy for LSS treatment, aims to retain at least 50% of the facet bilaterally and sufficient pars during facet-sparing laminotomy to preserve segmental stability (Lee et al., 2010a). Literature suggests that bilateral LOVE operations maintain similar segmental stability to unilateral procedures and are more accessible for surgeons compared to ULBD (Ho et al., 2015). However, both bilateral LOVE and Endo-LOVE operations may exhibit inadequate central canal decompression for severe central canal stenosis, despite their effectiveness in addressing lateral canal and recess stenosis (Jiang et al., 2023).

Endo-ULBD, a bilateral decompression protocol for endoscopic spinal treatment of LSS, stands out as a cutting-edge and highly sophisticated technique with excellent clinical efficacy. Nevertheless, clinical experience indicates several limitations of Endo-ULBD in treating severe and complex DLSS, such as insufficient intraoperative decompression of the central canal and contralateral recess, certain endoscopic “blind areas” in the contralateral field of view, and excessive facet joint resection leading to iatrogenic non-ischemic spondylolisthesis, among other factors. Additionally, when ULBD is performed ipsilaterally, the removal of excessive sagittal articular processes is more straightforward, especially when maximizing the enlargement of the spinal canal in severe DLSS(Kim et al., 2020).

Generally, Endo-ULBD initiates with ipsilateral laminotomy decompression, followed by contralateral canal decompression through a spinous process root breakthrough. While most contralateral facet joints can be preserved, central canal and lateral recess decompression might prove insufficient. In early cases, Choi et al. (Choi et al., 2016) reported that MRIs revealed that the ligamentum flavum after Endo-ULBD had not been adequately removed at several proximal and contralateral sites. In these patients, acute neurologic symptoms were resolved, but the affected lower leg fatigue remained. Occasional excessive facet joint resection is needed for lateral recess decompression, risking iatrogenic instability. Hua et al. (Hua et al., 2020a; Hua et al., 2020b) cautioned that adequate decompression without destabilizing joints might be a technical challenge for Endo-ULBD in cases with narrow interlaminar spaces, posterior marginal osteoproliferation of the vertebral body, and ossification of the posterior longitudinal ligaments. According to Wu et al., 2021, incomplete decompression was the main challenge in Endo-ULBD because of the limited surgical visibility, blind spots, and inadequate bony decompression. Endo-LOVE also faces similar challenges. The central canal decompression operation is prone to be blocked by the sagittal facet joints, which are needed to excise excessively. In many cases, the Endo-LOVE operation is just an idealized imagination. Endo-LOVE mainly focuses on the ipsilateral canal decompression. The lateral canal and recess can be enlarged ideally, but the complete resection of the facet joint is required. Sufficient decompression without violating the stability of the facet joints is technically tricky. Excessive facetectomy is inevitable for complete lateral recess decompression and foraminotomy in Endo-LOVE, which may ultimately exacerbate the postoperative instability. While facet hyperplasia and lateral recess stenosis support Endo-LOVE, severe central canal stenosis cases may experience inadequate decompression, leading to chronic neurogenic discomfort after acute symptom relief (Jiang et al., 2023).

To tackle these challenges, our proposed Cross-Overtop technique innovatively leverages the strengths of both Endo-ULBD and Endo-LOVE. The primary focus is on maximizing central canal enlargement while concurrently preserving facet joints and maintaining the integrity of the posterior complex. In practice, Cross-Overtop integrates and adapts Endo-ULBD and Endo-LOVE, using bilateral crossed trans-interlaminar laminotomy to enlarge the narrow central canal intersection. It targets complex DLSS cases characterized by the central spinal canal and bilateral recess stenosis, involving multiple factors like ligamentum flavum hypertrophy, intervertebral disc herniation, articular process hyperplasia, and nerve roots cohesion, or even mild spondylolisthesis. Among the DLSS pathological changes, posterior-center ligamentum flavum hypertrophy and articular process hyperplasia are commonly the main factors to be treated (Abbas et al., 2020). Thus, the hypertrophic ligamentum flavum resection is crucial for Cross-Overtop operation. Ligamentum flavum is most stout at the lumbar spine, extending from the lower and inner edges of the superior lamina to the upper and outer edges of the inferior lamina, participating in the composition of posterior and posterolateral walls surrounding the spinal canal, preventing excessive spinal flexion (Iwanaga et al., 2020). For Cross-Overtop, primary emphasis is placed on central spinal canal decompression, with a crucial observation being the anterior flexion constraint of the ligamentum flavum, as indicated by FE analysis. Extensive resection of ligamentum flavum and spinous process root may influence the posterior complex integrity, which is critical for stabilizing the lumbar motion segments, but the impact on biomechanics is unknown (Kwon et al., 2022). On the premise of the segmental stability verified in the study, Cross-Overtop has the advantages of avoiding redundant cauda equina nerve impaction and compensating for insufficient central canal decompression during Endo-ULBD and Endo-LOVE. It ensures complete central spinal canal enlargement without excessive facet joint damage, preserving posterior complex integrity and providing effective contralateral recess decompression while maintaining weight-bearing bony structures. The iatrogenic instability caused by excessive resection of the inferior articular process during ipsilateral decompression in conventional PEID can be effectively avoided. However, Cross-Overtop is more challenging to master technically and requires a longer learning curve. For example, ligamentum flavum hypertrophy might be necessary for coaxial endoscopic Cross-Overtop decompression, which minimizes the risk of dural sac damage during overtop operation. Whether Cross-Overtop or other PEID techniques, the primary DLSS treatment aim remains to relieve neurologic symptoms and enhance patient quality of life via dural sac and nerve root decompression. Adequate decompression yields better outcomes with spinal stability and normalized stress. Despite being a modified technique, systematic biomechanical research on Cross-Overtop is lacking.

FE analysis is one of the most important methods for biomechanical evaluation in spine surgery (Jia et al., 2020). It accurately captures the intricate biomechanics of the spine, enabling observation of column ROM and stress distribution across various structures. This study employed 3D FE models from CT images to evaluate operational effectiveness by comparing lumbar ROM and disc stress biomechanical properties. We developed an FE model based on normal body L4-L5 spinal CT images with varied parameters to simulate Endo-ULBD and Endo-LOVE, yielding theoretical data for virtual simulation. Previous biomechanical literature has shown no significant change in spinal stability after bilateral laminectomy (Rao et al., 2002; Tai et al., 2008). In the present study, Endo-LOVE, a bilateral laminectomy technique, showed consistent stability trends with the literature, affirming spinal stability post-surgery and validating the model. Meanwhile, Endo-ULBD exhibited comparable trends, suggesting its stability as well. This shows agreement with the Endo-ULBD surgery (Kim et al., 2020), which preserves the spinous process, contralateral lamina, and most of the posterior column in the clinic, which can further confirm the normative operation in the FE model. Because Endo-ULBD, Endo-LOVE, and Cross-Overtop all belong to the bilateral translaminar decompression procedures and have many anatomical operation similarities, we can overlap the former two mature operations within a specific range to ensure the regularity of the Cross-Overtop operation simulation in virtual FE model. Furthermore, the analyzed results made horizontal comparisons among the three endoscopic methods feasible. Hence, theoretical segmental stability and discal steady state post Cross-Overtop decompression are credible.

Validation has been a major problem in FE modeling of the spine. The predictive model needs to be modified with *in vivo* or *in vitro* measurements for further optimization. In neuromuscular FE analysis, where the complex system plays an important role in maintaining the physiological function, the practical application is indeed a difficult task (Esrafilian et al., 2022). We will conduct in-depth research in the future, such as resection of spinous processes and interspinous ligaments related to the laminectomy in open surgery, to broaden the scope of biomechanical study and demonstrate a higher value. In this study, several distinguishing characteristics of the repeated validation yield more convincing results. Firstly, the intact FE lumbar model and its parameters were used to validate virtual M0’s accuracy, laying the foundation for subsequent investigations. Furthermore, segmental properties of Endo-ULBD and Endo-LOVE were tested in the virtual FE model, bolstering PEID operational normality verification and Cross-Overtop’s regularization. Finally, the virtual finite element simulation serves as the theoretical foundation for clinical application. Conversely, real clinical cases offer more objective information to validate the aforementioned theoretical framework. The design of the operational scheme was rooted in virtual simulation theory, and its feasibility was subsequently demonstrated through actual clinical practice. ROM and disc stress were analyzed again in the actual FE models extracted from the real clinical data to compare with the theoretical results obtained from the virtual simulation data. The combination of theory and practice highlights the innovation and clinical value of the research, as well as the unexpected findings. Finally, both the virtual simulation and actual verification have their own merits, and the differences between the two FE models help to further reveal the truth of reality. In the research, the FE simulation model was built based on the ideal state with the normalized given parameters, whereas the practical FE real models were constructed according to the statistically calculated values. The spine position and local construction can be adjusted accordingly for the standard evaluation in the virtual FE analysis, while the real mean and standard deviation were used to decrease individual differences and systematic errors in the actual FE analysis. The comparative difference analysis makes sense in the related FE studies. For example, the stress increase of the intervertebral disc in the actual FE model was less than that in the virtual one, and this can be explained by the more stable lumbar segment in practically older and degenerative cases rather than that in theoretically younger and ordinary samples. This indicates that the disc at the surgical segment may actually be different from what virtual FE analysis predicted, and DLSS patients are less likely to experience accelerated disc degeneration after Cross-Overtop decompression in practice.

## 5 Conclusion

Cross-Overtop, as the modified endoscopic PEID technique, may, like the Endo-LOVE and ULBD techniques, potentially increase postoperative segmental mobility and disc stress, as validated within its stable range through FE modeling. A combined theoretical and practical analysis suggests that Cross-Overtop is unlikely to lead to severe adverse events such as intervertebral disc protrusion or lumbar segmental instability. Moreover, due to its simultaneous comprehensive expansion of the central vertebral canal volume and access to the contralateral foraminal region while maximizing the preservation of facet joints and posterior complex integrity, it can be recommended as an effective endoscopic treatment for severe DLSS.

## 6 Limitations of the study

The essence of finite element analysis is numerical calculation discretization, which yields the same results under equal conditions. Therefore, the research data derived from lumbar spine model only needs to be compared with the ROM of the human lumbar spine in previous literature studies, without the requirement of statistical analysis again. However, it is challenging to approximate the actual human condition because of individual patient differences. Additionally, this is a preliminary analysis of the clinical Cross-Overtop operation cases. The in-depth FE biomechanical evaluation of large samples must be carried out in the multi-center study.

## Data Availability

The raw data supporting the conclusion of this article will be made available by the authors, without undue reservation.
